# Thermostable Mismatch-Recognizing Protein MutS Suppresses Nonspecific Amplification during Polymerase Chain Reaction (PCR)

**DOI:** 10.3390/ijms14036436

**Published:** 2013-03-21

**Authors:** Kenji Fukui, Yoshitaka Bessho, Atsuhiro Shimada, Shigeyuki Yokoyama, Seiki Kuramitsu

**Affiliations:** 1 RIKEN SPring-8 Center, Harima Institute, 1-1-1 Kouto, Sayo-cho, Sayo-gun, Hyogo 679-5148, Japan; E-Mails: k.fukui@uic.osaka-u.ac.jp (K.F.); bessho@spring8.or.jp (Y.B.); 2 Department of Biological Sciences, Graduate School of Science, Osaka University, 1-1, Machikaneyama-cho, Toyonaka, Osaka 560-0043, Japan; E-Mail: a_shima@bio.sci.osaka-u.ac.jp; 3 RIKEN Systems and Structural Biology Center, 1-7-22 Suehiro-cho, Tsurumi, Yokohama 230-0045, Japan; E-Mail: yokoyama@riken.jp

**Keywords:** polymerase chain reaction, DNA mismatch repair, mismatch-recognizing protein, *Thermus thermophilus*, *Aquifex aeolicus*

## Abstract

Polymerase chain reaction (PCR)-related technologies are hampered mainly by two types of error: nonspecific amplification and DNA polymerase-generated mutations. Here, we report that both errors can be suppressed by the addition of a DNA mismatch-recognizing protein, MutS, from a thermophilic bacterium. Although it had been expected that MutS has a potential to suppress polymerase-generated mutations, we unexpectedly found that it also reduced nonspecific amplification. On the basis of this finding, we propose that MutS binds a mismatched primer-template complex, thereby preventing the approach of DNA polymerase to the 3′ end of the primer. Our simple methodology improves the efficiency and accuracy of DNA amplification and should therefore benefit various PCR-based applications, ranging from basic biological research to applied medical science.

## 1. Introduction

Since the polymerase chain reaction (PCR) was first conceptualized by Mullis in 1983 [1–3], the technique has become indispensable in a variety of fields ranging from basic biological research to applied medical science. As examples of its many uses, PCR-based amplification of specific sequences is essential for cloning genes of interest, detecting single nucleotide polymorphisms (SNPs) in diagnostic tests and identifying microbial infections.

Accurate amplification of nucleic acids by PCR is hindered by several technique-induced errors. This loss of fidelity could have dire consequences, especially in the field of medical sciences, where PCR may be used for genetic disease diagnosis, for example, on a single fertilized ovum. Therefore, improving the accuracy of PCR by suppressing errors is of crucial importance for human welfare. The two main types of PCR-associated errors are nonspecific amplification of oligonucleotides and polymerase-generated mutation. The former arises through mishybridization of primer sequences and the latter from misincorporation of deoxyribonucleotides by the DNA polymerase. Nonspecific amplification in PCR can be suppressed by adjusting the temperature of the annealing steps, by modulating Mg^2+^ concentration or by Hot-Start PCR; however, this is not always successful, especially when long DNAs are used as templates. Polymerase-generated errors can be reduced by using high-fidelity DNA polymerases. In reality, however, a compromise between replication accuracy and elongation efficiency is usually necessary [4,5], and many sequences cannot be efficiently amplified without the use of a low-fidelity DNA polymerase.

The reactions that result in both types of errors introduce mismatched bases that are specific targets for mismatch-recognizing proteins, such as MutS [6–10]; this observation suggests a possible mechanism by which such PCR-induced errors could be suppressed. MutS is an initiator of the DNA mismatch repair pathway and is conserved in almost all organisms, including a variety of thermophilic bacteria [11]. In 1995 and 1996, Wetmur issued two important US patents (5,877,280 and 6,294,325) [12,13] for the use of MutS proteins from hyperthermophiles, including *Thermus thermophilus* MutS (*Tth*MutS), to reduce the misincorporation of oligonucleotides and remove mismatched oligonucleotides from DNA mixtures during PCR. Since then, several studies have reported some success in removing mismatched oligonucleotides from DNA mixtures with MutS proteins. For example, in 1997, Smith and Modrich described the use of an *Escherichia coli* DNA mismatch repair system to remove error-containing sequences in DNA mixtures [14]. In 2005, Binkowski *et al*. established an error-filtering technique by using immobilized *Thermus aquaticus* MutS [15]. Although these techniques have advanced our ability to select error-free sequences in DNA populations, they are relatively intricate procedures and cannot be applied to simple PCR protocols. More recently, Mitani *et al*. (2007) used *T. aquaticus* MutS to suppress polymerase-generated mutations during isothermal DNA amplification [16]. This technique was shown to detect SNPs accurately, but it remained unclear whether it could be applied to PCR. Moreover, the possibility that MutS could suppress nonspecific amplification in addition to mutations during PCR had not been conceptualized at that time.

The purpose of this study was to establish a convenient error-suppressing technology for PCR that diminishes both nonspecific amplification and polymerase-produced mutations. The methods of Smith and Modrich [14] and Binkowski *et al*. [15] can be used to remove error-containing sequences after PCR is completed. By contrast, our method prevents amplification from mishybridized primers or mutated template sequences by simply adding a thermostable MutS protein to the reaction.

## 2. Results and Discussion

**Figure 1:**
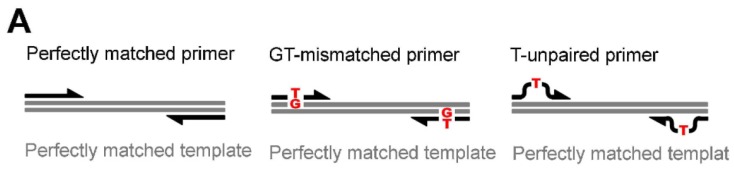

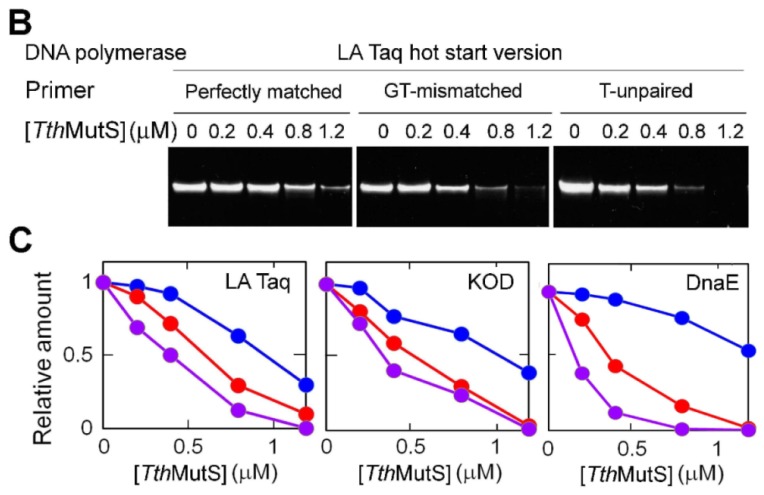
Effects of *Tth*MutS on standard polymerase chain reaction (PCR) amplification of an 80-bp template. (**A**) Schematic representation of the primers and templates used in (**B**,**C**). Perfectly matched, GT-mismatched or unpaired T-containing primers were used; (**B**) Perfectly matched (left), GT-mismatched (middle) or unpaired T-containing (right) primers were used to amplify the perfectly matched template; (**C**) The relative amounts of the products from perfectly matched (blue), GT-mismatched (red) or unpaired T-containing (purple) primers were plotted against the *Tth*MutS concentration for reactions using three polymerases: LA Taq (left), KOD polymerase (middle) and *A. aeolicus* DnaE (right). The amounts of the products were normalized by those at 0 μM *Tth*MutS.

To investigate the error-suppressing capability of MutS, we constructed a system to monitor the amplification efficiency of an 80-base pair (bp) template DNA (Supplementary Figure S1A) after 20 cycles of amplification, the midpoint of the PCR amplification curve (Supplementary Figure S1B). First, we determined whether *Tth*MutS [17] could suppress nonspecific amplification caused by mishybridization of primers (Figure 1A). Two types of mismatches were investigated; mispaired bases, such as GT, and unpaired bases, such as single-base insertion/deletion loops [6,7]. As representatives of such mispaired and unpaired bases, we used primers containing a GT mismatch or an unpaired T, respectively. In the absence of *Tth*MutS, the mismatched primers had no effect on the amplification efficiency in standard PCR. By contrast, amplification from both types of mismatched primers was specifically suppressed in the presence of *Tth*MutS (Figure 1B,C). High concentrations of *Tth*MutS slightly reduced amplification from perfectly matched primers. This may be explained by the fact that *Tth*MutS also binds nonspecifically to DNA; therefore, nonspecific binding to the template DNA at the extension step could interfere with the progression of DNA polymerases (as discussed later). As shown in Figure 1C, *Tth*MutS suppressed amplification by LA Taq, which contains *T. aquaticus* DNA polymerase I [18], an A family DNA polymerase. We confirmed that *Tth*MutS was also effective with members of the two other DNA polymerase families; KOD polymerase [19] (B family) (Figure 1C, middle panel) and *Aquifex aeolicus* DnaE [20] (DNA polymerase III α subunit) (C family) (Figure 1C, right panel), indicating that *Tth*MutS-dependent error suppression is effective with a wide range of DNA polymerases.

**Figure 2:**
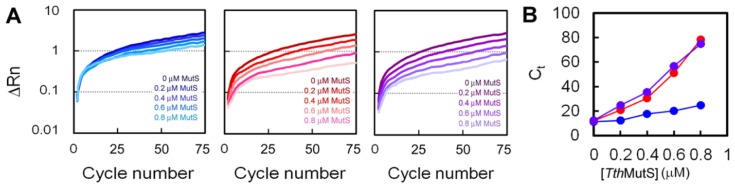
Effects of *Tth*MutS on amplification of an 80-bp template by using real-time PCR. (**A**) An 80-bp perfectly matched dsDNA was amplified by AmpliTaq Gold DNA polymerase by using perfectly matched (left), GT-mismatched (middle) or unpaired T-containing (right) primers in the presence of 0, 0.2, 0.4, 0.6, 0.8 or 1.0 μM *Tth*MutS. The change in fluorescence (ΔRn) was plotted against the cycle number; (**B**) The threshold cycle (*C*_t_) was determined and plotted against the *Tth*MutS concentration. Blue, red and purple circles indicate amplification by using perfectly matched, GT-mismatched and unpaired T-containing primers, respectively. All experiments were repeated three times. Bars indicate standard deviations.

We also examined the effect of *Tth*MutS on the amplification of the 80-bp template DNA by using real-time PCR and monitoring the increase in fluorescence of SYBR Green I, which specifically stains double-stranded (ds) DNA (Figure 2A). The threshold cycle for each amplification was determined and plotted against the concentration of *Tth*MutS in the reaction (Figure 2B). The results clearly showed that *Tth*MutS suppressed amplification from the mismatched primers more effectively than from the perfectly matched primers.

As a control, we tested the *A. aeolicus* MutL *C*-terminal domain [21], which is heat stable at 95 °C, but lacks mismatch-recognition ability (Supplementary Figure S2). This protein had no effect on template amplification with the mismatch-containing primers, indicating that the error-suppressing activity of *Tth*MutS on nonspecific amplification is due to mismatch recognition.

**Figure 3:**
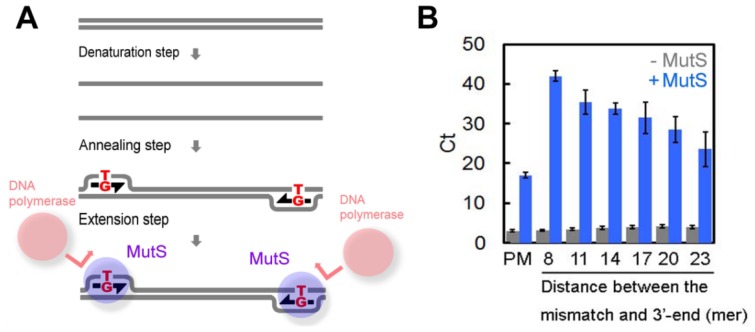
Model for *Tth*MutS suppression of mismatched primer-dependent amplification. (**A**) Schematic representation of the model. Mishybridization of primers at the annealing step generates mismatched bases that are tightly bound by MutS, which block the approach of DNA polymerases to the 3′ end of the primers; (**B**) The 80-bp perfectly matched template was amplified by AmpliTaq Gold DNA polymerase by real-time PCR using mismatched primers. The threshold cycle (*C*_t_) was determined and plotted against the distance between the mismatched bases and 3′ end of the primer (8, 11, 14, 17, 20 or 23 bp). PM, perfectly matched primer. Blue and gray bars indicate experiments with or without 1.0 μM *Tth*MutS, respectively. Experiments were repeated three times. Bars indicate standard deviations.

These results demonstrate that *Tth*MutS suppresses nonspecific amplification during PCR. We propose a model in which *Tth*MutS binds to the mismatched primer-template complex at the extension step, thereby blocking the binding of the DNA polymerase to the 3′ end of the primer (Figure 3A). If this model is correct, we would expect the error-suppressive effect of *Tth*MutS to be reduced by increasing the distance between the mismatch-bound *Tth*MutS and the 3′ end of the primer. Indeed, we found this to be the case. As shown in Figure 3B, the effect of *Tth*MutS was reduced in proportion to the number of nucleotides between the mismatch and 3′ end of the primers, substantiating our hypothesis. It should be noted that we cannot exclude the possibility that *Tth*MutS also prevents elongation to the 5′ end of the template, generating incompletely extended DNA fragments, which leads to unstable hybridization between the primers and the template at the next cycle.

**Figure 4:**
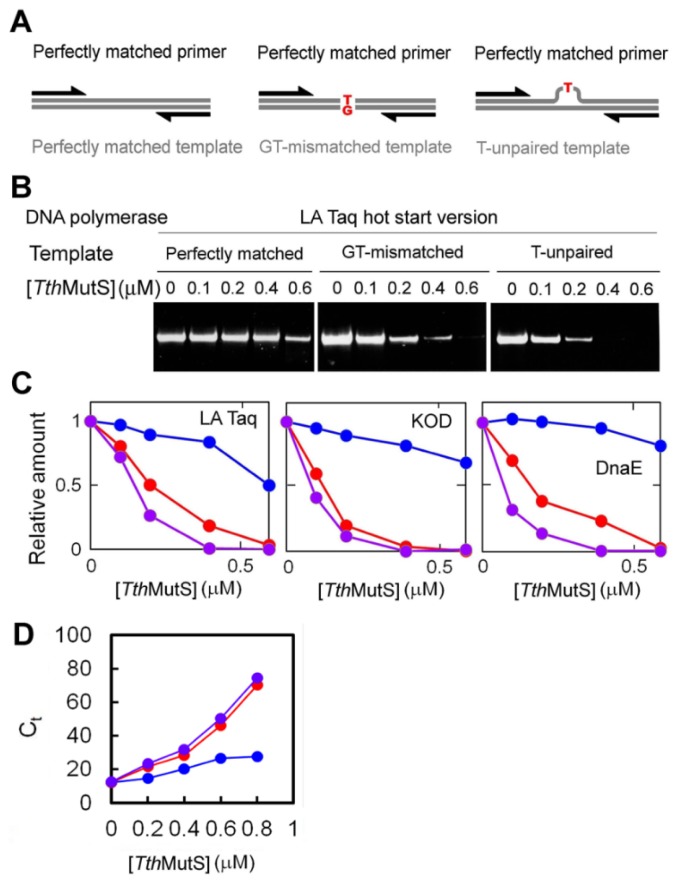
Effects of *Tth*MutS on standard PCR amplification of mismatched templates. (**A**) Schematic representations of the primers and templates used in (**B**,**C**); (**B**) A perfectly matched primer was used to amplify perfectly matched (left), GT-mismatched (middle) or unpaired T-containing (right) templates; (**C**) Relative amounts of amplified fragments from reactions using perfectly matched (blue), GT-mismatched (red) or unpaired T-containing (purple) templates were plotted against the *Tth*MutS concentration. The amounts of the products were normalized by those at 0 μM *Tth*MutS; (**D**) The effect of *Tth*MutS on amplification of the 80-bp mismatched template was examined by real-time PCR. The perfectly matched (blue), GT-mismatched (red) and unpaired T-containing (purple) templates were amplified by AmpliTaq Gold DNA polymerase by using perfectly matched primers. The threshold cycle (*C*_t_) was determined and plotted against the *Tth*MutS concentration. Experiments were repeated three times. Bars indicate standard deviations.

We next tested the ability of MutS to inhibit amplification from GT-mismatched or T-unpaired templates (Figure 4A). We found that mismatches in the template did not affect amplification efficiency in the absence of *Tth*MutS, but the addition of *Tth*MutS resulted in mismatch-specific suppression of amplification (Figure 4B,C). The effect of *Tth*MutS was observed for all three DNA polymerase family proteins (Figure 1C), and as expected, amplification was not affected by the *A. aeolicus* MutL *C*-terminal domain, which lacks mismatch-recognition capability (Supplementary Figure S3). We also monitored amplification of the perfectly matched and mismatched 80-bp templates by real-time PCR to evaluate the effect of *Tth*MutS on the threshold cycle (Figure 4D). *Tth*MutS specifically increased the threshold cycle for the amplifications from mismatched templates, which showed good concordance with the results of the standard PCR experiments.

**Figure 5:**
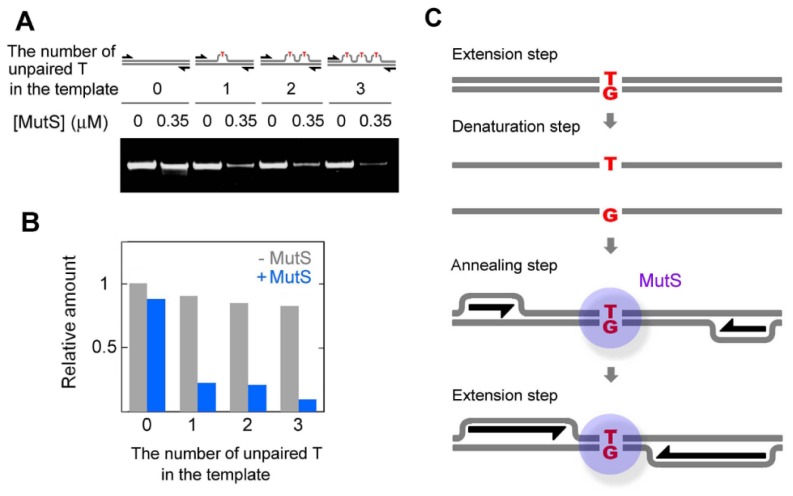
Effect of the number of mismatches in an 80-bp template on amplification efficiency in the presence of *Tth*MutS. (**A**) Templates containing zero, one, two or three unpaired T mismatches were used. PCR was performed using LA Taq Hot-Start version with perfectly matched primers in the absence or presence of 0.35 μM *Tth*MutS; (**B**) Quantification of the relative amounts of amplified fragments. Gray and blue columns indicate amplification in the absence or presence of *Tth*MutS, respectively. The amounts of the products were normalized by that from perfectly-matched template at 0 μM *Tth*MutS; (**C**) Model for the mechanism by which *Tth*MutS suppresses amplification from mismatched templates.

*Tth*MutS suppressed amplification of templates containing one, two or three mismatches with equal efficiency (Figure 5A,B), indicating that a single mismatch is sufficient for *Tth*MutS-dependent suppression; a possible mechanism is illustrated in Figure 5C. The template mismatches are uncoupled at the denaturation step, but are regenerated at the annealing step, where they are recognized by *Tth*MutS. Binding of *Tth*MutS then interferes with the progression of DNA polymerase that accompanies strand displacement [16,22,23]. This mechanism is analogous to that previously proposed by Mitani *et al*. (2007) for isothermal amplification. Another possible mechanism for the suppression of amplification from mismatched templates is that *Tth*MutS may stabilize the dsDNA during the denaturing step. The stabilized templates may then fail to denature and be unavailable for further amplification. However, we found contrasting results by examining thermal denaturation of dsDNA by a dissociation curve analysis in real-time PCR. In these experiments, the addition of *Tth*MutS did not affect thermal denaturation of perfectly matched, GT-mismatched or unpaired T-containing 21-bp dsDNA (Supplementary Table S1).

**Figure 6:**
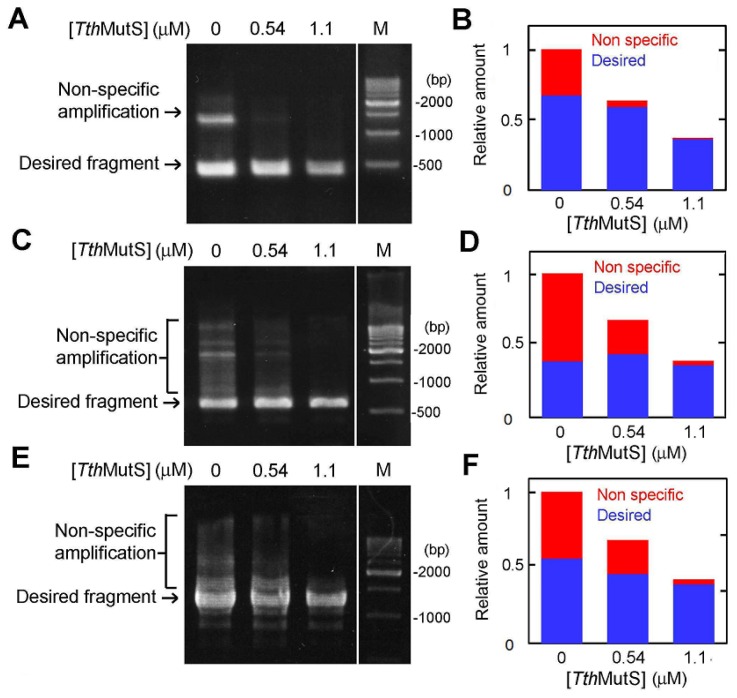
Effects of MutS on gene amplification from genomic DNA. (**A**) A 423-bp region of *ttha1806* of *T. thermophilus* was amplified in the presence of *Tth*MutS. The 500-bp DNA ladder is indicated by the lane marked “M”. A second aliquot of *Tth*MutS was added to the reaction at the end of the fifteenth cycle. The final concentration of *Tth*MutS is shown at the top of the lanes; (**B**) The relative amounts of nonspecific and desired amplification products from (A) are shown in red and blue, respectively; (**C**) Amplification of the 594-bp region of *tthb071* in the presence of *Tth*MutS; (**D**) Quantification of the amplification products from (C); (**E**) The 1278-bp region of *ttha1548* was amplified; (**F**) Quantification of the amplification products shown in (E). The amounts of the products were normalized by the amount of total products at 0 μM *Tth*MutS.

To determine whether *Tth*MutS is effective in error suppression during PCR amplification of longer templates, we amplified three different sequences (423-, 594- and 1278-bp) from *T. thermophilus* genomic DNA using Takara LA Taq and perfectly matched primers (Figure 6A–F). To allow mishybridization of primers, the annealing step was performed at 48 °C, which is a relatively low temperature (see Methods Section). Nonspecific amplification was readily detected under these PCR conditions and in the absence of *Tth*MutS. However, the addition of *Tth*MutS to the reactions significantly improved the amplification specificity (Figure 6). Because *Tth*MutS is not stable at temperatures above 82 °C (Supplementary Figure S4), we supplemented the reaction with additional *Tth*MutS after completion of the fifteenth cycle of the 30-cycle program. Our results show that 1.1 μM *Tth*MutS was sufficient to suppress nonspecific amplification. In the same experiments, we also tested the thermostable *A. aeolicus* DNA-binding protein MutL [24], which binds nonspecifically to DNA, but does not recognize base pair mismatches. However, we found that *A. aeolicus* MutL suppressed both specific and nonspecific amplifications (Supplementary Figure S5). Although *A. aeolicus* MutL has weak endonuclease activity, DNA degradation was not apparent under the PCR reaction conditions employed (Supplementary Figure S6). *A. aeolicus* MutL exhibits approximately 10- and 100-fold higher affinity for perfectly matched dsDNA than does *Tth*MutS and *A. aeolicus* MutL *C*-terminal domain (CTD), respectively [21]. The strong suppression of specific amplification by *A. aeolicus* MutL (Supplementary Figure S5) could be due to its nonspecific DNA-binding activity, which is of much higher affinity than that of *Tth*MutS (*K*_d_ values are 0.15 μM and 2 μM, respectively). This explains why *A. aeolicus* MutL showed much stronger suppression of specific amplification than *Tth*MutS. These results indicate that mismatch recognition is essential for the selective suppression of nonspecific amplification by *Tth*MutS.

**Figure 7:**
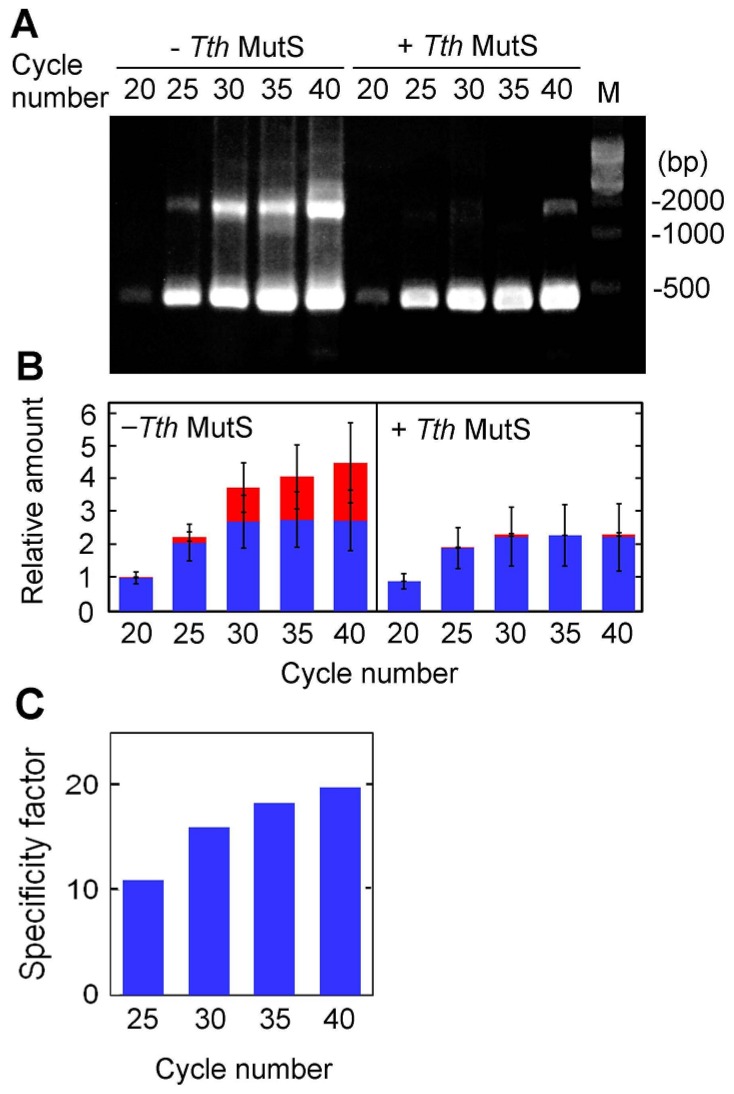
PCR cycle-dependence of the amplification of a 423-bp region of *ttha1806*. (**A**) The amplification was performed in the presence or absence in 1.1 μM *Tth*MutS and it was terminated at the end of 20, 25, 30, 35 or 40 PCR cycles. The 500-bp DNA ladder is indicated by the lane marked “M”; (**B**) The relative amounts of nonspecific and desired amplification products from (A) are shown in red and blue, respectively. The experiments were performed three times. Bars indicate standard deviations. The amounts of the products were normalized by the amount of total products at the end of twentieth cycle in the absence of *Tth*MutS; (**C**) Specificity factor for 1.1 μM *Tth*MutS at each PCR cycle.

We next examined the PCR cycle-dependence of *Tth*MutS error-suppressing activity by amplifying the 423-bp sequence for 20, 25, 30, 35 or 40 cycles in the presence of 1.1 μM *Tth*MutS (Figure 7A,B). We observed that *Tth*MutS effectively suppressed nonspecific amplification at each cycle analyzed. To quantify the effect of *Tth*MutS, we introduced the specificity factor, which is the ratio between the rate of suppression of nonspecific and specific amplification (see “Online Methods” for details). For the PCR cycle numbers tested with the 423-bp sequence, the estimated specificity factors of 1.1 μM *Tth*MutS were between 10 and 20, as shown in Figure 7C. These estimates suggest that amplification was ~10–20-fold more specific in the presence of *Tth*MutS than in its absence.

**Figure 8:**
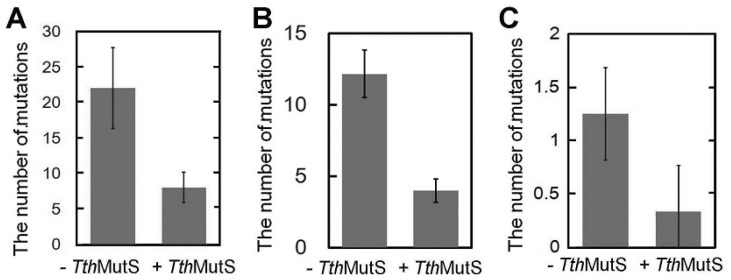
*Tth*MutS suppressed DNA polymerase-generated mutations during PCR. (**A**) A 423-bp fragment of *ttha1806* was amplified in the presence of 200 μM MnCl_2_ with or without 1.1 μM *Tth*MutS, cloned into the pT7Blue vector and sequenced using the T7 promoter primer. The numbers of mutations in the fragment are shown; the values represent the mean ± standard deviation for five independent experiments; (**B**) A 423-bp fragment of *ttha1806* was amplified in the presence of 200 μM MnCl_2_ and 400 μM adenylyl imidodiphosphate (AMPPNP) with or without 1.1 μM *Tth*MutS, cloned into the pT7Blue vector and sequenced using the T7 promoter primer; (**C**) *ttha1548* was amplified in the absence of MnCl_2_, cloned into the pT7Blue vector and sequenced using the T7 promoter and U-19-mer primers. Mutations in the 5′- and 3′-terminal 500 bp-regions of *ttha1548* were counted.

Having demonstrated the ability of *Tth*MutS to suppress nonspecific PCR amplification, we next examined its effect on DNA polymerase-generated mutations. For this, we amplified the 423-bp genomic fragment and cloned and sequenced the PCR products. To facilitate detection of a *Tth*MutS effect, PCR was performed with LA Taq under error-prone conditions in the presence of 200 μM MnCl_2_. Our sequencing analysis showed that addition of 1.1 μM *Tth*MutS reduced the frequency of polymerase-generated mutations by approximately two-thirds (Figure 8A). Because MutS has weak ATPase and dATPase activities, we cannot exclude the possibility that the addition of *Tth*MutS perturbs the dNTP balance, leading to misincorporation by DNA polymerases. To test this, we added equal concentrations of dATP and adenylyl imidodiphosphate (AMPPNP), which inhibits the ATP/dATPase activity of *Tth*MutS. The addition of AMPPNP had no effect on the reduction of polymerase-generated mutations by *Tth*MutS (Figure 8B). This result suggests that the dATPase activity of *Tth*MutS is weak and has little or no effect on the balance of dNTPs. A similar reduction in the polymerase-generated mutation rate was obtained when the experiment was performed in the absence of MnCl_2_ (Figure 8C).

**Figure 9:**
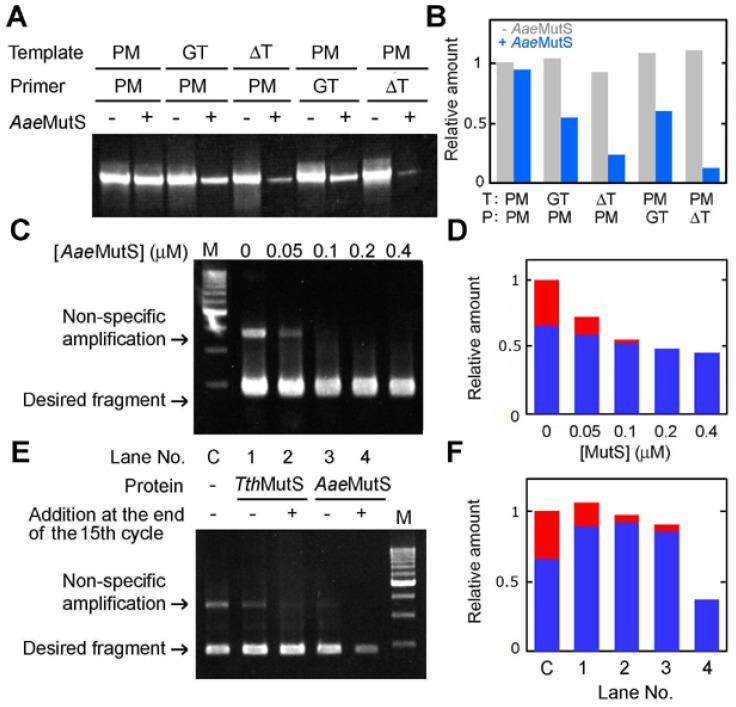
Effects of *Aae*MutS on amplification of an 80-bp template. (**A**) Results of amplification of an 80-bp template in the presence of 0.2 μM *Aae*MutS. PM, GT and ΔT indicate perfectly matched, GT-mismatched and unpaired T-containing primers or templates, respectively; (**B**) Quantification of the relative amounts of PCR products from (**A**); (**C**) The same experiment as in Figure 6A was performed using *Aae*MutS. The amounts of the products were normalized by that from perfectly-matched template and primers at 0 μM *Aae*MutS; (**D**) Quantification of the relative amounts of nonspecific (red) and desired (blue) amplification products in (**C**). The amounts of the products were normalized by the amount of total products at 0 μM *Aae*MutS; (**E**) The *ttha1806* gene was amplified in the presence of 0.3 μM *Tth*MutS or *Aae*MutS. At the end of the fifteenth cycle, 0 or 0.3 μM *Tth*MutS or *Aae*MutS was added to each reaction tube. Control amplification in the absence of *Tth*MutS or *Aae*MutS is indicated as “C”; (**F**) Quantification of the amplification products from (**E**). The amounts of the products were normalized by the amount of total products at 0 μM *Aae*MutS.

Because *Tth*MutS is not stable above ~85 °C, we sought to improve the method by preparing MutS from the hyperthermophilic bacterium *A. aeolicus*, which can grow at temperatures of up to 95 °C [25]. *A. aeolicus* MutS (*Aae*MutS) was successfully overexpressed, purified and shown to be stable at 95 °C (Supplementary Figure S7). Of note, *Aae*MutS was able to suppress error-containing amplification of an 80-bp oligonucleotide (Figure 9A,B) and a genomic DNA template (Figure 9C,D). *Aae*MutS at a final concentration of 0.2–0.4 μM was sufficient to obtain mismatch-specific suppression. Importantly, *Aae*MutS exhibited a strong suppressive effect without the need to supplement the reaction with an additional protein at the end of the fifteenth cycle, as we had observed for *Tth*MutS (Figure 9E,F).

**Figure 10:**
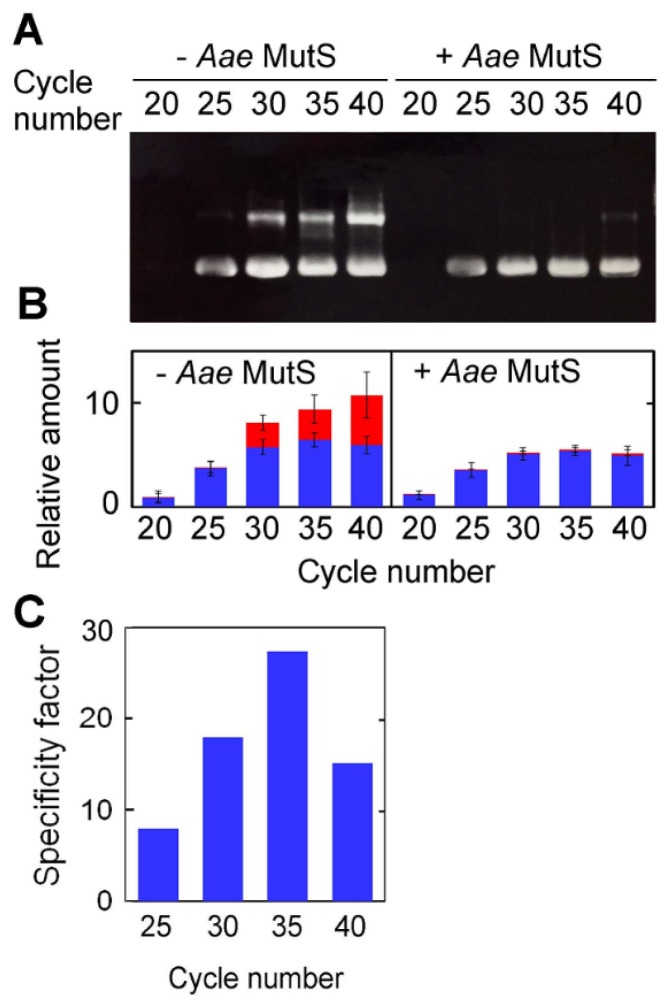
The PCR cycle-dependence of the amplification of a 423-bp region of *ttha1806* in the presence or absence of *Aae*MutS. (**A**) The same experiment as in Figure 7 was performed with 0 or 0.3 μM *Aae*MutS; (**B**) The relative amounts of nonspecific and desired amplification products from (A) are shown in red and blue, respectively. The experiments were performed three times. Bars indicate standard deviations. The amounts of the products were normalized by the amount of total products at the end of twentieth cycle in the absence of *Aae*MutS; (**C**) Specificity factor for 0.3 μM *Aae*MutS at each PCR cycle.

The PCR cycle-dependence of *Aae*MutS activity was tested by amplification of the 423-bp sequence over 20–40 cycles in the presence or absence of 0.4 μM *Aae*MutS (Figure 10A,B). The specificity factors for 0.4 μM *Aae*MutS under these conditions were between 10 and 30 (Figure 10C), which was very similar to those of *Tth*MutS.

**Figure 11:**
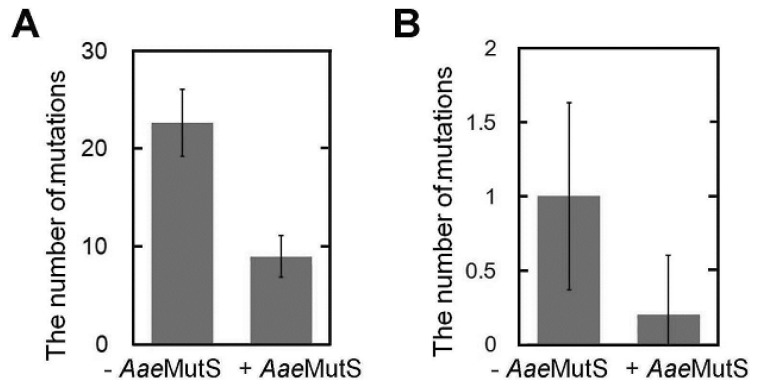
*Aae*MutS suppressed DNA polymerase-generated mutations during PCR. (**A**) The same experiment as in Figure 8A was performed using 0.3 μM *Aae*MutS; (**B**) The same experiment as in Figure 8C was performed using *Aae*MutS.

Sequencing of PCR products showed that 0.3 μM *Aae*MutS decreased the number of polymerase-generated mutations to approximately 30% of the control level (Figure 11A,B). From these results, we conclude that addition of *Aae*MutS is a convenient and useful method for suppressing both nonspecific amplification and polymerase-generated errors in PCR.

The major novel finding in this study is that MutS can suppress nonspecific amplification; that is, amplification from mismatched primers. Although we expected that MutS would be able to suppress amplification from a mismatched template, our results clearly demonstrate that MutS suppresses amplification from both mismatched templates and primers. Furthermore, by preparing MutS from the extremely thermophilic bacterium *A. aeolicus*, we also showed that the error-reducing effects of MutS can be applied to standard PCR conditions.

## 3. Experimental Section

### 3.1. Overexpression and Purification of Proteins

The *A. aeolicus mutS* gene was amplified by PCR using *A. aeolicus* genomic DNA as the template. The forward and reverse primers were 5′-CCATGGAGAAATCTGAGAAAGAGCTCAC-3′ and 5′-AGATCTTTATTAAGCTCCGGACTCCTTTTT-3′, respectively, which contained NcoI and BglII sites, respectively (underlined). The amplified fragment was ligated into the NcoI and BamHI sites of pET-HisTEV [26] to yield a pET-HisTEV/*A. aeolicus mutS* plasmid. Sequence analysis revealed that the construction was error free.

*E. coli* BL21(DE3) (Novagen, Madison, WI, USA) was transformed with pET-HisTEV/*A. aeolicus mutS* and cultured at 37 °C in 1.5 L of YT medium [0.8% (*w*/*v*) tryptone, 0.5% (*w*/*v*) yeast extract and 0.5% (*w*/*v*) NaCl] containing 50 μg/mL ampicillin. When the culture density reached 4 × 10^8^ cells/mL, isopropyl β-d-thiogalactopyranoside was added to a final concentration of 100 μM to induce expression. The cells were grown at 37 °C for a further 4 h, harvested by centrifugation and lysed by sonication in buffer I (20 mM Tris-HCl and 50 mM NaCl, pH 7.8). The sonicate was heated to 70 °C for 10 min, centrifuged at 48,000× *g* for 20 min, and the supernatant was then loaded onto a Talon resin column (40 mL; Clontech, Palo Alto, CA, USA) equilibrated with buffer I. The column was washed with 300 mL of buffer I and then eluted with buffer I containing 150 mM imidazole. The fractions containing *Aae*MutS were detected by sodium dodecyl polyacrylamide gel electrophoresis (SDS-PAGE) and collected, and (NH_4_)_2_SO_4_ was added to the fraction to a final concentration of 1 M. The solution was loaded onto a Toyopearl-Phenyl column (Tosoh, Tokyo, Japan) equilibrated with buffer I containing 1 M (NH_4_)_2_SO_4_. The column was washed with 100 mL of buffer I containing 1 M (NH_4_)_2_SO_4_ and then eluted with a 300 mL gradient of 1 to 0 M (NH_4_)_2_SO_4_ in buffer I. The fractions containing *Aae*MutS were detected by SDS-PAGE and concentrated with a Vivaspin concentrator (Vivascience, Göttingen, Germany). The protein solution was dialyzed against 20 mM Tris-HCl and 50 mM NaCl (pH 7.8).

The *A. aeolicus dnaE* gene, which encodes a DNA polymerase III α subunit, was amplified by PCR using *A. aeolicus* genomic DNA as the template. The forward and reverse primers used were 5′-ACATATGAGTAAGGATTTCGTCCACCTTCA-3′ and 5′-AGGATCCTTATTAAATTATGACC TTCACTCCCAG-3′, respectively, which contained NdeI and BamHI sites, respectively (underlined). The fragment was ligated into the NdeI and BamHI sites of pET-HisTEV to generate a pET-HisTEV/*A. aeolicus dnaE* plasmid. Sequence analysis revealed that the construction was error free.

*E. coli* Rosetta 2(DE3) (Novagen) was transformed with pET-HisTEV/*A. aeolicus dnaE*. The cells were cultured and harvested using the same procedure as described above for *A. aeolicus mutS* overexpression. The product of *A. aeolicus dnaE*, *Aa*eDnaE, was purified using the same procedure as described for *Aae*MutS.

*Tth*MutS and the *C*-terminal domain of *A. aeolicus* MutL were overexpressed and purified as previously described [17,21].

### 3.2. Circular Dichroism Spectrometry

Circular dichroism measurements were carried out with a Jasco spectropolarimeter (J-720W; Jasco, Tokyo, Japan). All measurements were performed using a 0.1-cm cell at 25 °C. The residue molar ellipticity [θ] was defined as 100θ_obs_/*lc*, where θ_obs_ was the observed ellipticity, *l* the length of the light path in centimeters and *c* the residue molar concentration of each protein. The measurements were performed in a solution consisting of 50 mM potassium phosphate (pH 7.0), 20 mM NaCl and 5 μM *Aae*MutS or *Tth*MutS.

### 3.3. PCR Using an 80-bp DNA Template

DNAs were synthesized by BEX Co (Tokyo, Japan). The 80-mer single-stranded DNA 5′-GGTAAGCGACATCTCTCTCGAGTGATACGTCCTAGCAGAATTCCGCTACATGAAGCTT CTAGAGGTTACTGCAGCCTGAC-3′ was annealed to 5′-GTCAGGCTGCAGTAACCTCTAGAAG CTTCATGTAGCGGAATTCTGCTAGGACGTATCACTCGAGAGAGATGTCGCTTACC-3′ to obtain the perfectly matched template, 5′-GTCAGGCTGCAGTAACCTCTAGAAGCTTCATGTAG CGGTATTCTGCTAGGACGTATCACTCGAGAGAGATGTCGCTTACC-3′ to obtain the GT-mismatched template and 5′-GGTAAGCGACATCTCTCTCGAGTGATACGTCCTAG CAGATTCCGCTACATGAAGCTTCTAGAGGTTACTGCAGCCTGAC-3′ to obtain the unpaired T-containing template. The perfectly matched, GT-mismatched and unpaired T-containing forward and reverse primers were 5′-GTCAGGCTGCAGTAAC-3′ and 5′-CGTAAGCGACATCTC-3′, 5′-GTCAGGTTGCAGTAAC-3′ and 5′-GGTAAGTGACATCTC-3′ and 5′-GTCAGGCTTGCAGTAA C-3′ and 5′-GGTAAGCTGACATCTC-3′, respectively. PCR was performed with 0.06 units/μL LA Taq Hot-Start Version (Takara, Shiga, Japan), 0.03 units/μL KOD polymerase (Toyobo, Tokyo, Japan) or 40 nM *A. aeolicus* DnaE, in 1× Takara LA PCR buffer II (Takara, Shiga, Japan) containing 20 nM template DNA; 400 nM primers; 400 μM dATP, dTTP, dCTP and dGTP and various concentrations of *Tth*MutS or *Aae*MutS. Twenty PCR cycles were run using the temperature controlling system PC707 (Astec, Tokyo, Japan): denaturation step, 80 °C for 1 min; annealing, 48 °C for 1 min; and extension, 70 °C for 2 min (slope of 1 min). The abnormally low annealing temperature was used to permit extension of mismatched primers, which may explain the low efficiency of the PCR amplification in these experiments. At the end of the program, 6 μL of the reaction solution was mixed with 1 μL of sample buffer (50% (*v*/*v*) glycerol and 0.05% (*w*/*v*) bromophenol blue) and electrophoresed on a 9% polyacrylamide gel in 1× TBE buffer (89 mM Tris-borate and 2 mM EDTA). The gel was stained with SYBR Gold (Invitrogen, Carlsbad, CA, USA), and the products were detected under ultraviolet light at 254 nm. The amplified DNA products were quantified using ImageJ software (National Institutes of Health, Bethesda, MD, USA).

To determine whether MutS performance was affected by the number of template mismatches, 80-bp DNAs were prepared by hybridizing 5′-GTCAGGCTGCAGTAACCTCTAGAAGCTTCATGT AGCGGAATTCTGCTAGGACGTATCACTCGAGAGAGATGTCGCTTACC-3′ to either 5′-GGTAAGCGACATCTCTCTCGAGTGTATACGTCCTAGCAGATTCCGCTACATGAAGCTTC TAGAGGTTACTGCAGCCTGAC-3′ or 5′-GGTAAGCGACATCTCTCTCGAGTGTATACGTCCT AGCAGATTCCGCTACATGAAGCTTCTGAGGTTACTGCAGCCTGAC-3′, to generate templates carrying 2 or 3 unpaired T residues, respectively.

### 3.4. Real-Time PCR

Aliquots (25 μL) of SYBR Green Master Mix (Applied Biosystems, Foster City, CA, USA), containing AmpliTaq Gold DNA polymerase, were mixed with an equal volume of a solution containing 2 nM 80-bp template dsDNA and 400 nM primers in the presence of various concentrations of *Tth*MutS. Real-time PCR was performed with the 7300 Real-Time PCR system (Applied Biosystems). The reaction solution was heated at 50 °C for 10 min and 90 °C for 5 min to activate the AmpliTaq DNA polymerase, followed by 80 cycles: denaturation step, 80 °C for 1 min; annealing, 48 °C for 1 min; and extension, 70 °C for 2 min. The perfectly matched, GT-mismatched and unpaired T-containing primers were 5′-GTCAGGCTGCAGTAAC-3′ and 5′-CGTAAGCGACATCTC-3′, 5′-GTCAGGTTGCAGTAAC-3′ and 5′-GGTAAGTGACATCTC-3′ and 5′-GTCAGGCTTGCAGT AAC-3′ and 5′-GGTAAGCTGACATCTC-3′, respectively. Melting curve analysis confirmed the specificity of amplification with each primer and template set (data not shown). The data were analyzed, as previously described [27]. The primers with varying distances (8, 11, 14, 17, 20 and 23 nucleotides) between the mismatch and 3′ end of the primer were as follows: 5′-GTCAGGCTTGCAGTAAC-3′a and 5′-GGTAAGCTGACATCTC-3′, 5′-GTCAGGCTTGCA GTAACCTC-3′ and 5′-GGTAAGCTGACATCTCTCT-3′, 5′-GTCAGGCTTGCAGTAACCTCTAG-3′ and 5′-GGTAAGCTGACATCTCTCTCGA-3′, 5′-GTCAGGCTTGCAGTAACCTCTAGAAG-3′ and 5′-GGTAAGCTGACATCTCTCTCGAGTG-3′, 5′-GTCAGGCTTGCAGTAACCTCTAGAAGCTT-3′ and 5′-GGTAAGCTGACATCTCTCTCGAGTGATA-3′ and 5′-GTCAGGCTTGCAGTAACCTCT AGAAGCTTCAT-3′ and 5′-GGTAAGCTGACATCTCTCTCGAGTGATACGT-3′, respectively.

### 3.5. PCR Using Genomic DNA Template

The 5′-terminal 423-bp region of *ttha1806* was amplified using the primers 5′-GAGACCA CCCGTAGGCGGCT-3′ and 5′-CTTAAGGGGCCTCGCGCTCT-3′; a 594-bp region of *tthb071* was amplified using 5′-CGTCAGGCTGGCCTTCCCCCTTTCC-3′ and 5′-TTCCAGTGGCGGTCGTA GACCCCGTC-3′; a 1278-bp region of *ttha1548* was amplified using 5′-GAGGAGGTGCTCTA CGTGGGCAAGGCC-3′ and 5′-GGGAAGGTCCTTGAGGCTTCCCGTGTAGC-3′; and a 1515-bp region of *ttha1645* was amplified using 5′-ATATCATATGCGTGACGTCCTCGAGGTCC-3′ and 5′-ATATAGATCTTTATTACTCGAGCCTCTCCAGAAGGGCCTC-3′. The reactions were performed in 1× Takara GC I buffer (Takara) containing 0.06 units/μL LA Taq (Takara); 5 ng/μL *T. thermophilus* HB8 genomic DNA; 400 nM primers; 100 μM CoCl_2_; 400 μM dATP, dTTP, dCTP and dGTP and various concentrations of *Tth*MutS or *Aae*MutS. Thirty PCR cycles were run using an Astec OC707 thermal cycler. Conditions for amplification of *ttha1806* and *ttha1645* were as follows: denaturation step, 95 °C for 1 min; annealing, 58 °C for 1 min; and extension, 70 °C for 2 min. Amplification conditions for *tthb071* and *ttha1548* were as follows: denaturation step, 95 °C for 1 min; annealing, 50 °C for 1 min; and extension, 70 °C for 2 min. For reactions containing *Tth*MutS, a second aliquot of *Tth*MutS solution (equal to the amount in the initial reaction mixture) was added at the end of the fifteenth cycle. At the end of the program, 10 μL of reaction solution was mixed with 1 μL of loading buffer [50% (*v*/*v*) glycerol, 0.9% (*w*/*v*) SDS and 0.05% (*w*/*v*) bromophenol blue] and electrophoresed on a 1.5% agarose gel in 1× TBE buffer. The gel was stained with ethidium bromide, and the products were visualized under ultraviolet light at 254 nm. DNA products were quantified using ImageJ software. The “specificity factor” was defined as the ratio between the rate of suppression of nonspecific amplification and the rate of specific amplification and obtained using the following equation:

Specificity factor at *n* μM MutS = *A*_N0_*A*_Sn_/*A*_Nn_*A*_S0_, where *A*_N0_, *A*_Sn_, *A*_Nn_ and *A*_S0_ are the amounts of nonspecific amplification at 0 μM MutS, specific amplification at *n* μM MutS, nonspecific amplification at n μM MutS and specific amplification at 0 μM MutS, respectively.

For the experiments examining polymerase-induced mutations, PCR reactions were performed using *ttha1806* and *ttha1548* templates in the presence of 0 or 200 μM MnCl_2_. The reaction products were ligated into the pT7Blue vector (Novagen, Madison, WI, USA) by using the TA cloning technique and sequenced using a BigDye terminator version 3.1 cycle sequencing kit (Applied Biosystems, Foster City, CA, USA).

## 4. Conclusions

Our new methodology offers a significant improvement over current PCR-related technologies. The suppression of nonspecific amplification will be important for a wide range of PCR-based technologies, including the detection of viral and bacterial infections and identification of SNPs. The observed reduction in polymerase-generated mutations (~65%) is also significant. For example, cloning a gene (typically 1000 bp) with a DNA polymerase that has low fidelity, but high elongation efficiency generally produces more than 1 mutation per 1000 bp (Figures 8C and 11B). In such cases, the addition of MutS offers an attractive option to enable error-free recovery of the gene.

A thermostable RecA protein has previously been used to suppress nonspecific amplification during PCR, although this protein was unable to suppress polymerase-generated errors [28,29]. RecA promotes the correct hybridization of primers and template sequences, thereby reducing the frequency of mishybridizations. Thus, the mechanism by which MutS suppresses nonspecific amplification is quite different from that of RecA, suggesting that the combined use of MutS and RecA may allow even greater accuracy in PCR-based applications. Nucleic acid analogs, such as locked nucleic acids, peptide nucleic acids and morpholino oligomers have also been used as PCR probes to sustain the specificity of amplification [30]. The MutS-based technique described here could also be used in combination with such nucleic acid analogs. Further studies will be required to determine whether MutS can recognize a mismatch comprising a natural base and an analog.

## Supplementary Information
