# Supplementary material for: Thermostable Mismatch-Recognizing Protein MutS Suppresses Nonspecific Amplification during Polymerase Chain Reaction (PCR)
Source: Int J Mol Sci. 2013 Mar 21;14(3):6436–53. doi: 10.3390/ijms14036436 (PMC3634412; doi:10.3390/ijms14036436)
Supplement: Supplementary file 1 [file ijms-14-06436-s001.pdf]

Supplementary Information

Figure S1. PCR of 80-bp perfectly-matched template DNA using perfectly-matched primers. (**A**) Lane1: Reaction mixture without DNA polymerase was loaded. Lane 2: Reaction mixture with LA Taq hot start vesion was loaded. Lane 3: Reaction mixture with LA Taq hot start version was digested with EcoRI, and then loaded. The EcoRI site is located at the center of the template DNA; (**B**) PCR was stopped at the end of each cycel, and the reaction mixture was analyzed by PAGE. The amount of amplified fragment was quantified by ImageJ software and plotted against the cycle number. The amounts of the products were normalized by that at the end of 30th cycle.

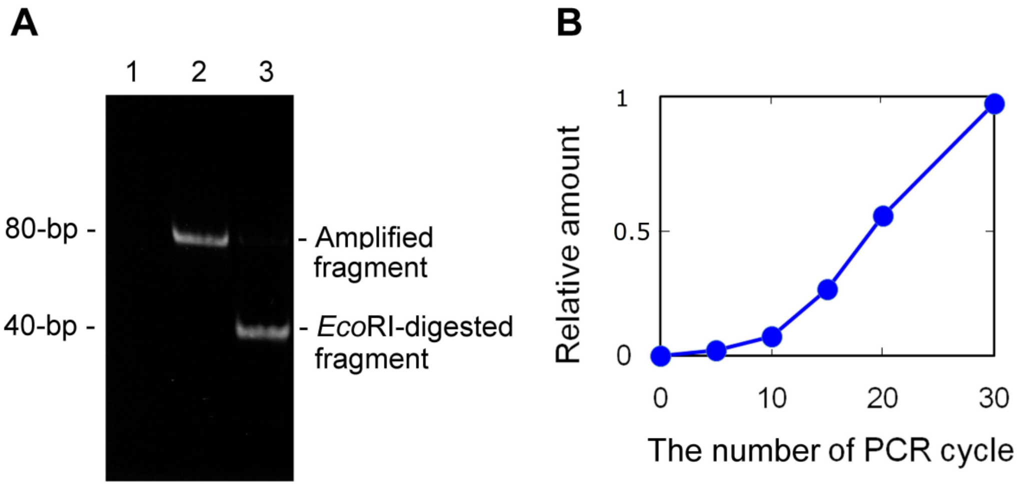

Figure S2. A thermostable protein with no mismatch-recognition ability did not suppress mismatched primer-dependent amplification. (**A**) PCR of an 80-bp perfectly-matched template was performed with perfectly matched (left) or unpaired T-containing (right) primers in the presence of the *C*-terminal domain (CTD) of A. aeolicus MutL; (**B**) The relative amounts of the amplified fragments were quantified and plotted against *A. aeolicus* MutL CTD concentration. The results from the perfectly-matched and unparired T-containing primers are indicated by the blue and purple lines, respectively. The amounts of the products were normalized by that at 0 M MutL CTD.

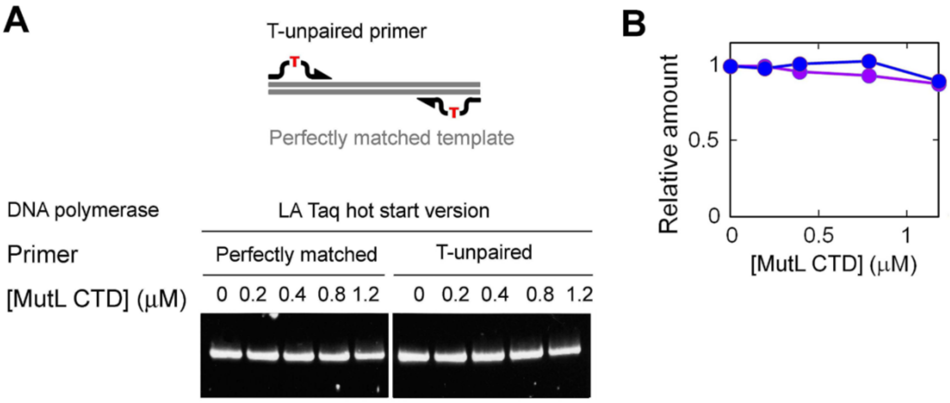

Figure S3. The thermostable A. aeolicus MutL CTD, which has no mismatch-recognizing ability, did not suppress mismatched template-dependent amplification. (**A**) Amplification products of 80-bp perfectly-matched (left) or unpaired T-containing (right) template was performed using perfectly-matched primers in the presence of A. aeolicus MutL CTD; (**B**) The relative amounts of amplified fragments were quantified and plotted against concentration of A. aeolicus MutL CTD.

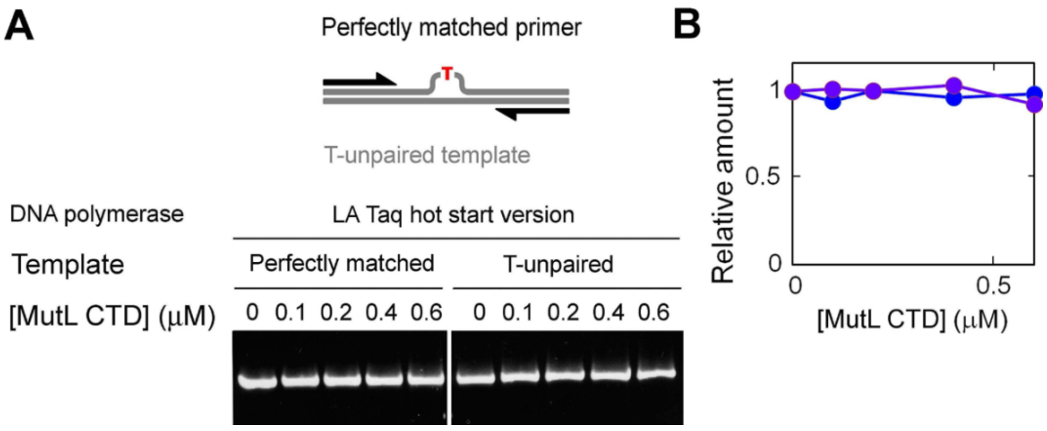

Figure S4. CD measurement of TthMutS. (**A**) The far-ultraviolet CD spectrum of TthMutS; (**B**) Temperature dependence of residue molar ellipticity at 222 nm. A heating rate of 1 °C/min was used.

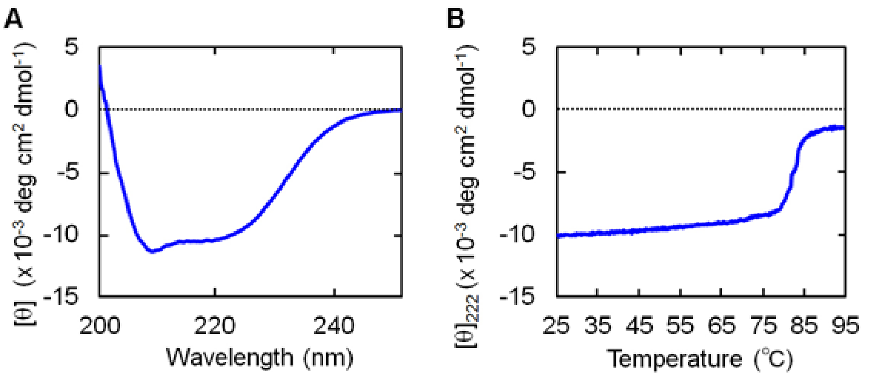

Figure S5. Effect of A. aeolicus MutL on amplification of a 423-bp region of ttha1806. The *ttha1806* gene from the *T. thermophilus* HB8 genome was amplified by PCR using perfectly-matched primers. (**A**) Amplification of a 423-bp region of *ttha1806*. The concentrations of *A. aeolicus* MutL are shown at the top of the panel; (**B**) The relative amounts of non-specific and specific amplification products in (**A**) were determined and are shown as red and blue columns, respectively. The amounts of the products were normalized by that of the total products at 0 M MutL.

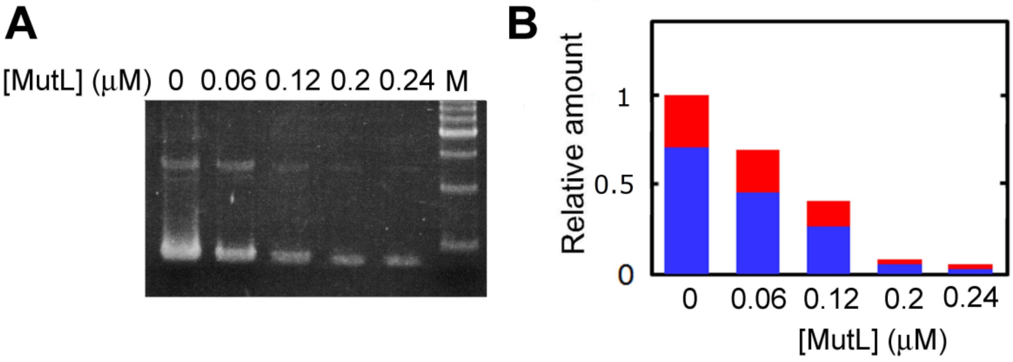

Figure S6. The endonuclease activity of *A. aeolicus* MutL was negligible under the PCR reaction condition. (**A**) A 594-bp PCR product was purified and reacted with 0.5 M *A. aeolicus* MutL under the same reaction condition as Figure S5A. Reaction solutions were subjected to 1.5% agarose gel electrophoresis in 1× TBE buffer. The gel was stained with ethidium bromide and the DNA fragments were visualized under ultraviolet light at 254 nm. Reaction period is shown at the top of the panel; (**B**) The relative amount of the undegraded DNA fragment was quantified by imageJ software and plotted against reaction time. The amounts of the DNA fragments were normalized by that at the 0 min reaction time.

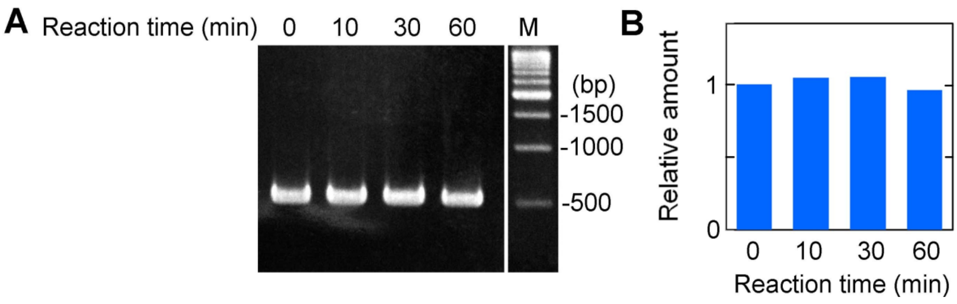

Figure S7. CD measurement of AaeMutS. (**A**) The far-ultraviolet CD spectrum of AaeMutS; (**B**) Temperature dependence of residue molar ellipticity at 222 nm. A heating rate of 1 °C/min was used.

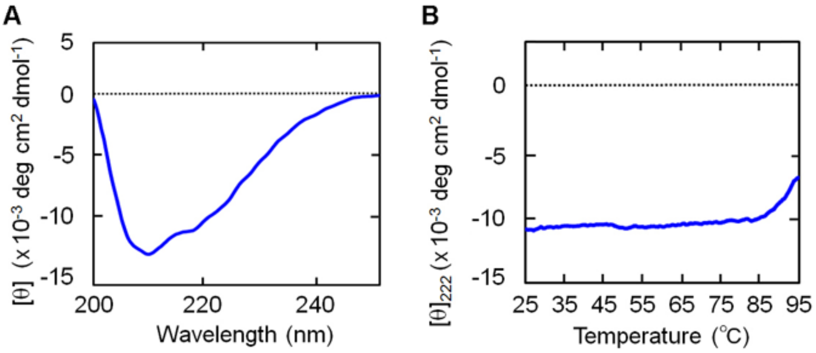

Table S1. The melting temperature of 21-bp perfectly-matched, GT-mismatched, and unpaired T-containing double-stranded DNAs. The 21-bp double-stranded DNA (0.1 M) was incubated with 1 M TthMutS in 50 mM HEPES (pH 7.5), 100 mM KCl and 1 mM $MgCl_2$ at 37 °C for 30 min. Then, SYBR Green I (Applied Biosystems) was added and thermal denaturation step was monitored by dissociation program of 7300 Real-Time PCR system (Applied Biosystems).

Sequence	Mismatch	*Tth*MutS	Melting temperature (°C)
5'-CTTGACTATGTCCGCTCTACG-3'<br>3'-GAACAGATACAGGCGAGATGC-5'	None	-	61.4
5'-CTTGACTATGTCCGCTCTACG-3'<br>3'-GAACAGATACAGGCGAGATGC-5'	None	+	61.2
5'-CTTGACTATG**T**CCGCTCTACG-3'<br>3'-GAACTGATAC**G**GGCGAGATGC-5'	GT	-	58.9
5'-CTTGACTATG**T**CCGCTCTACG-3'<br>3'-GAACTGATAC**G**GGCGAGATGC-5'	GT	+	58.1
5'-CTTGACTATG**T**CCGCTCTACG-3'<br>3'-GAACAGATAC-GGCGAGATGC-5'	Unpaired T	-	58.3
5'-CTTGACTATG**T**CCGCTCTACG-3'<br>3'-GAACAGATAC-GGCGAGATGC-5'	Unpaired T	+	58.1

© 2013 by the authors; licensee MDPI, Basel, Switzerland. This article is an open access article distributed under the terms and conditions of the Creative Commons Attribution license (http://creativecommons.org/licenses/by/3.0/).
